# Towards a definitive symptom structure of obsessive−compulsive disorder: a factor and network analysis of 87 distinct symptoms in 1366 individuals

**DOI:** 10.1017/S0033291720005437

**Published:** 2022-10

**Authors:** Matti Cervin, Euripedes C. Miguel, Ayşegül Selcen Güler, Ygor A. Ferrão, Ayşe Burcu Erdoğdu, Luisa Lazaro, Sebla Gökçe, Daniel A. Geller, Yasemin Yulaf, Şaziye Senem Başgül, Özlem Özcan, Koray Karabekiroğlu, Leonardo F. Fontenelle, Yankı Yazgan, Eric A. Storch, James F. Leckman, Maria Conceição do Rosário, David Mataix-Cols

**Affiliations:** 1 Department of Clinical Sciences Lund, Lund University, Lund, Sweden; 2 Department of Psychiatry, Faculdade de Medicina FMUSP, Universidade de Sao Paulo, Sao Paulo, SP, Brazil; 3 Department of Psychology, Beykent University, Istanbul, Turkey; 4 Department of Clinical Medicine (Neurosciences), Porto Alegre Health Sciences Federal University, Porto Alegre, Brazil; 5 Department of Child and Adolescent Psychiatry, Marmara University, Istanbul, Turkey; 6 Department of Child and Adolescent Psychiatry and Psychology, Hospital Clínic, IDIBAPS, CIBERSAM, University of Barcelona, Barcelona, Spain; 7 Department of Child and Adolescent Psychiatry, Maltepe University, Istanbul, Turkey; 8 Department of Psychiatry, Massachusetts General Hospital, Harvard Medical School, Boston, Massachusetts, USA; 9 Department of Psychology, Gelişim University, Istanbul, Turkey; 10 Department of Psychology, Hasan Kalyoncu University, Gaziantep, Turkey; 11 Department of Child and Adolescent Psychiatry, İnönü University, Malatya, Turkey; 12 Department of Child and Adolescent Psychiatry, Ondokuz Mayıs University, Samsun, Turkey; 13 Turner Institute for Brain and Mental Health, Monash University, Victoria, Australia; 14 D'Or Institute for Research and Education (IDOR) and Institute of Psychiatry, Federal University of Rio de Janeiro, Rio de Janeiro, Brazil; 15 Güzel Günler Clinic, Istanbul, Turkey; 16 Yale Child Study Center, New Haven, CT, USA; 17 Department of Psychiatry and Behavioral Sciences, Baylor College of Medicine, Houston, TX, USA; 18 Departments of Psychiatry, Pediatrics & Psychology, Child Study Center, Yale University, New Haven, CT, USA; 19 Department of Psychiatry, Federal University of São Paulo (UNIFESP), Brazil; 20 Department of Clinical Neuroscience, Centre for Psychiatry Research, Karolinska Institutet, Stockholm, Sweden; 21 Health Care Services, Region Stockholm, Stockholm, Sweden

**Keywords:** Obsessive–compulsive disorder, symptom dimensions, heterogeneity, factor analysis, network analysis

## Abstract

**Background:**

The symptoms of obsessive−compulsive disorder (OCD) are highly heterogeneous and it is unclear what is the optimal way to conceptualize this heterogeneity. This study aimed to establish a comprehensive symptom structure model of OCD across the lifespan using factor and network analytic techniques.

**Methods:**

A large multinational cohort of well-characterized children, adolescents, and adults diagnosed with OCD (*N* = 1366) participated in the study. All completed the Dimensional Yale-Brown Obsessive−Compulsive Scale, which contains an expanded checklist of 87 distinct OCD symptoms. Exploratory and confirmatory factor analysis were used to outline empirically supported symptom dimensions, and interconnections among the resulting dimensions were established using network analysis. Associations between dimensions and sociodemographic and clinical variables were explored using structural equation modeling (SEM).

**Results:**

Thirteen first-order symptom dimensions emerged that could be parsimoniously reduced to eight broad dimensions, which were valid across the lifespan: Disturbing Thoughts, Incompleteness, Contamination, Hoarding, Transformation, Body Focus, Superstition, and Loss/Separation. A general OCD factor could be included in the final factor model without a significant decline in model fit according to most fit indices. Network analysis showed that Incompleteness and Disturbing Thoughts were most central (i.e. had most unique interconnections with other dimensions). SEM showed that the eight broad dimensions were differentially related to sociodemographic and clinical variables.

**Conclusions:**

Future research will need to establish if this expanded hierarchical and multidimensional model can help improve our understanding of the etiology, neurobiology and treatment of OCD.

## Introduction

Phenotypic validity is crucial for identifying causal and maintaining mechanisms in mental disorders (Insel et al., 2010). Obsessive−compulsive disorder (OCD) poses challenges in this respect because of its heterogeneous symptoms spanning a broad array of fears, thoughts, emotions, urges, and behaviors (Leckman & Bloch, 2008). Empirical studies have suggested that this heterogeneity can be organized under several partially overlapping symptom dimensions, with some uncertainty about the exact number or nature of these dimensions. The only meta-analysis to date suggested four symptom dimensions: forbidden thoughts, hoarding, symmetry, and contamination/cleaning (Bloch, Landeros-Weisenberger, Rosario, Pittenger, & Leckman, 2008). These dimensions are temporally stable (Fernandez de la Cruz et al., 2013; Mataix-Cols et al., 2002) and related to differences in neural substrates (Mataix-Cols et al., 2004; van den Heuvel et al., 2009) and heritability (Iervolino, Rijsdijk, Cherkas, Fullana, & Mataix-Cols, 2011; Lopez-Sola et al., 2016). However, other dimensional models have been suggested, with some evidence suggesting that the forbidden thoughts factor may best be split into two separate factors entailing symptoms of responsibility/harm and sexual/religious concerns, respectively (Miguel et al., 2008; Torres et al., 2016). Further, it is currently unclear whether a similar symptom structure of OCD is present across the lifespan, which is important because childhood-onset OCD may constitute a somewhat different patient group (Leckman et al., 2010).

To date, research on the symptom structure of OCD has primarily relied on the symptom checklist of the Yale-Brown Obsessive−Compulsive Scale (Y-BOCS) (Goodman et al., 1989), which includes a limited number of symptom types derived from the considerable clinical experience of the developers. Most research employing the Y-BOCS has been carried out using binary coding (i.e. present/absent) of a limited number of broad symptom categories rather than individual symptoms included under each category, possibly obscuring more complex symptom structures. The few studies that have employed more fine-grained item-level analyses have yielded inconsistent results (ranging from three to six dimensions), but sample sizes have generally been small, inadequate statistical techniques used, and miscellaneous symptoms often excluded (see Cameron et al. (2019) for a review).

The Dimensional Y-BOCS (DY-BOCS) was developed nearly two decades after its predecessor with the primary aim to better capture the symptom heterogeneity of OCD. In addition to providing a global OCD severity score, like the Y-BOCS, the DY-BOCS also allows for the scoring of six theoretically derived symptom dimensions (Rosario-Campos et al., 2006). Further, the DY-BOCS includes an expanded symptom checklist of 88 specific symptoms which includes a comprehensive list of miscellaneous symptoms (not readily grouped under the five theory-based dimensions), descriptions of mental rituals and avoidance across OCD dimensions, and fuller descriptions of symptoms related to symmetry and forbidden thoughts. Thus, the DY-BOCS symptom checklist is the most comprehensive instrument currently available to help further understand the symptom structure of OCD.

Through international collaboration, we gathered a uniquely large cohort of well-characterized individuals with OCD who had DY-BOCS item-level data. We applied state-of-the-art factor and network analytic techniques to shed further light on the most accurate and parsimonious way to conceptualize the heterogeneous symptom nature of OCD. In a series of exploratory analyses, we examined unique associations between the resulting symptom dimensions and a range of key sociodemographic and clinical variables.

## Methods

### Sample

Data from 1366 children, adolescents, and adults with a confirmed diagnosis of OCD from four countries were pooled for analysis. A large Brazilian sample (*n* = 1001) was included alongside separate child samples from Brazil (*n* = 81), Spain (*n* = 95), and Turkey (*n* = 142), and an adult sample from the United Kingdom (*n* = 47). All samples included treatment-seeking individuals with OCD. Sociodemographic and clinical characteristics of the participants are presented in online Supplementary Material Table S1. All studies were conducted in accordance with the Declaration of Helsinki and were approved by ethical review boards at each site. Participants provided written informed consent (or assent if under the age of 18).

### Measures

#### DY-BOCS

The 88 individual symptom items of the DY-BOCS symptom checklist are rated as currently present/formerly present/absent. In this study, the currently present ratings were used to indicate the presence of a symptom. One miscellaneous item that cuts across several other symptoms (‘*Avoidance to prevent any of these miscellaneous obsessions and compulsions’*) was omitted, resulting in a total of 87 items for analysis. The checklist is followed by a clinician-led interview assessing symptom severity across six theoretically derived dimensions (aggressive, religious/sexual, symmetry, contamination, hoarding, and miscellaneous symptoms) and of overall OCD severity. The DY-BOCS has sound psychometric properties and good construct validity in youth [see summary in Cervin *et al*. (2019a, 2019b)] and adult samples (Pertusa, Fernandez de la Cruz, Alonso, Menchon, & Mataix-Cols, 2012; Rosario-Campos *et al*. 2006), but the factor structure of its symptom checklist has never been investigated (Rosario-Campos et al., 2006).

#### Demographic and clinical variables

For a large subset of participants (*N* = 1001; Brazilian sample), comprehensive sociodemographic and clinical data were collected within the Brazilian Research Consortium on Obsessive−Compulsive Spectrum Disorders (BRC-OCSD) study, described in detail elsewhere (Miguel et al., 2008). The following self-report scales from BRC-OCSD were used: Beck Anxiety Inventory (anxiety symptoms), Beck's Depression Inventory (depressive symptoms), and Brown Assessment of Beliefs Scale (insight). Clinician-collected BRC-OCSD data on suicidality, family history of OCD and tic disorders, age at OCD symptom onset, OCD course, and severity of sensory phenomena were also used. Last, diagnostic and Y-BOCS data from BRC-OCSD and Y-BOCS data from the UK sample were used. Descriptive statistics for all included variables are presented in online Supplementary Material Table S2.

### Statistical analysis

#### Planned analyses

Confirmatory factor analysis (CFA) was used to test whether the theoretically derived six-factor structure of the DY-BOCS would exhibit a good fit to the data. For comparison purposes, we also tested a model in which a single, general OCD factor explained correlations among symptoms. Model fit was evaluated using χ^2^, confirmatory fit index (CFI), root mean square error of approximation (RMSEA), standardized mean square residual (SRMR), and Tucker−Lewis fit index (TLI). Adequate model fit is indicated by a lower χ^2^ value, higher CFI/TLI (values >0.90 are indicative of adequate fit), and lower RMSEA and SRMR (values <0.06 and 0.08, respectively, are indicative of good fit) (Schermelleh-Engel, Moosbrugger, & Müller, 2003). If model fit was adequate for the six-factor model, we set out to explore parsimonious ways to model the factors using second- and potentially higher-order factors. We also wanted to examine whether the model had good fit in subsamples of participants (children/adults; men/women; country of origin). Diagonally weighted least-squares estimation was used and robust fit indices computed. The overall proportion of missing data was small (0.64%) and pairwise deletion was employed for all analyses. All CFAs were run with *lavaan* in R Studio.

#### Planned follow-up analysis

If model fit for the two tested models were inadequate, we planned to use 40% of the full sample to run an exploratory factor analysis (EFA) to establish an empirically derived factor structure that could then be tested, using CFA, in the remaining 60% of the sample. Again, if we were able to establish an adequate factor structure, more parsimonious models would be explored.

#### Network analysis of resulting symptom dimensions

The covariance structure of the latent variables resulting from the best-fitting symptom dimension model were used to reproduce underlying case-level data using the R-package *MASS*. These data were used to estimate a partial correlation network. Because of potential problems with collinearity, we set out to not include latent variable pairs that were highly correlated. Thus, the most parsimonious dimensional solution was used as input for the network model. Regularization with EBICglasso was used, through which the magnitude of all variable-to-variable associations are shrunk so that spurious associations are set to zero (Epskamp & Fried, 2018). The force-directed Fruchterman−Reingold algorithm was used to plot the network, placing dimensions with many and strong unique associations to other dimensions centrally and pairs of strongly associated dimensions closely. To compute and compare the degree to which each dimension was associated with other dimensions in the network, we estimated expected influence, which is a measure of each dimension's positive associations to other dimensions (Robinaugh, Millner, & McNally, 2016). Confidence intervals around network parameters were estimated by running 1000 bootstraps and results used to test whether there were statistically significant differences in expected influence for the different dimensions (α level = 0.05). The full statistical script is available as a Supplementary file.

#### Sociodemographic and clinical associations

To examine associations between symptom dimensions and sociodemographic and clinical factors, we fitted regression models within a structural equation modeling (SEM) framework. In these models, the latent symptom dimensions from the best-fitting symptom structure model were the independent variables, and the sociodemographic/clinical variables were the dependent variables. Thus, we accounted for covariance among symptom dimensions, resulting in unique associations between each symptom dimension and the sociodemographic/clinical variable. One clinical variable of interest was a general factor of psychopathology (*p* factor). The *p* factor is thought to reflect a broad vulnerability to symptoms across the psychiatric spectrum regardless of the severity of any specific psychiatric dimension or disorder (Kotov et al., 2017). In accordance with this definition, we estimated *p* within the SEM model using lifetime history for the following 11 diagnostic classes as indicators: any depressive disorder, any anxiety disorder, any psychosis spectrum disorder, any eating disorder, any bipolar spectrum disorder, body dysmorphic disorder (BDD), skin picking disorder (SPD), trichotillomania, any substance addiction disorder, ADHD, and illness anxiety disorder (IAD). To adjust for multiple testing, associations with an α level <0.01 were considered statistically significant in the SEM regression models.

## Results

### Planned confirmatory and follow-up EFA

The theory-derived six-factor model of the DY-BOCS exhibited a poor fit in the full sample, as did the single-factor model (see Table 1). In line with our statistical plan, we randomly split the sample into two groups (40/60% of the total sample) and performed an EFA based on the first 40% (*n* = 547) to derive an empirically supported factor structure. The mean age in the EFA sample was 30.7 years (s.d. = 15.1), 52% were female, 24% were children/adolescents, and 80% were from Brazil. A tetrachoric correlation matrix was computed; no symptoms correlated above 0.75. The overall Kaiser−Meyer−Olkin (KMO) test value was 0.86 with no single value being under 0.50 and Bartlett's test of sphericity was significant (*p* < 0.0001). Thus, the data were well suited for EFA and all 87 items were included. Horn's parallel analysis (used to determine the number of factors to retain in the first stage of an EFA) suggested 13 factors. Principal axis factoring and promax rotation was used to extract these factors. The 13 factors explained 44.2% of the variance in the full set of variables. Loadings for all items and the proportion of participants in the full sample that endorsed each item are presented in online Supplementary Material Table S3.

**Table 1.** Fit indices for the different models tested with DY-BOCS symptom data

		χ^2^	*df*	*p*	CFI	TLI	RMSEA	SRMR
DY-BOCS models								
1	Theory-derived six-factor structure (CFA in full sample)	9822.9	3725	<0.001	0.874	0.871	0.035	0.098
2	Single-factor model (CFA in full sample)	23 442.9	3740	<0.001	0.594	0.585	0.062	0.146
3	EFA derived 13-factor structure (CFA in 60% of sample)	5018.8	3491	<0.001	0.943	0.941	0.023	0.086
4	Model 3 but with a single incompleteness factor (CFA in 60% of sample)	5364.7	3514	<0.001	0.931	0.929	0.025	0.091
5	Model 3 but with a single forbidden thoughts factor (CFA in 60% of sample)	5135.7	3503	<0.001	0.940	0.937	0.024	0.088
6	Model 3 but with a single contamination factor (CFA in 60% of sample)	5344.1	3503	<0.001	0.932	0.929	0.025	0.091
7	**Model 5 with three second-order factors and a general factor (CFA in 60% of sample)**	**5295.5**	**3554**	**<0.001**	**0.935**	**0.934**	**0.024**	**0.095**
8	Model 5 with three second-order factors and a general factor (CFA in full sample)	7100.4	3554	< 0.001	0.926	0.924	0.027	0.086

DY-BOCS, dimensional Yale-Brown obsessive compulsive scale; CFA, confirmatory factor analysis; EFA, exploratory factor analysis; OCD, obsessive−compulsive disorder; χ^2^, chi-squared; *df*, degrees of freedom; CFI, comparative fit index; TLI, Tucker−Lewis Index; RMSEA, root mean square error of approximation; SRMR, standardized root mean square residual.

Best-fitting model, because of the principle of parsimony, highlighted in bold.

### Confirmatory factor analyses and planned exploration of more parsimonious solutions

The mean age in the CFA sample (*n* = 821; 60% of the full sample) was 29.7 years (s.d. = 14.9), 57% were female, 27% were children/adolescents, and 79% were from Brazil. Model fit of the 13-factor structure in this sample was adequate (Table 1), all indicators loaded significantly (*p* < 0.001) onto its modeled factor, and 71% of all items had a standardized loading >0.70. Only one item had a standardized loading <0.50 (‘*Skin picking’*). The mean standardized loading was >0.60 for all factors and >0.80 for 9 out of the 12 factors. Standardized factor loadings, factor names, item content, and proportion of participants endorsing at least one symptom within each dimension are presented in Table 2. We fitted the model separately in men and women, Brazilian and European participants, and children and adults. Fit indices were overall adequate in all subsamples and are presented in online Supplementary Material Table S4.

**Table 2.** Item content, factor names, standardized factor loadings, and proportion of participants endorsing at least one symptom within each category of the 13-factor DY-BOCS model

Harm/Checking		Accuracy		NJR behaviors		Dirt/Cleaning	
1. I have obsessions that I might harm myself	0.76	1. I have obsessions about things needing to be perfect/exact/”just-right’.	0.85	1. I need to repeat routine activities	0.63	1. I am obsessed with dirt or germs	0.86
2. I have obsessions that I will be harmed	0.71	2. I have obsessions about symmetry	0.77	2. I have counting compulsions	0.59	2. I am overly concerned or disgusted with bodily waste or secretions	0.76
3. I check that I did not harm myself or was not harmed	0.67	3. I check that I did not make mistakes	0.89	3. I have compulsions that involve symmetrical touching and/or evening-up behaviors	0.69	3. I have obsessions about insects or animals	0.81
4. I have obsessions that I might harm other people	0.76	4. I re-read or re-write things	0.86	4. I need to touch, tap, or rub things	0.65	4. I am bothered by sticky substances or residues	0.72
5. I have obsessions that I will harm other people without meaning to hurt them	0.83	5. I have ordering or arranging compulsions.	0.79	5. I have mental rituals other than checking or evening-up	0.69	5. I am concerned I will get ill because of contamination	0.84
6. I have obsessions that I may be responsible for something else terrible happening	0.67	6. I fear not saying ‘just the right thing’	0.75	6. I have eating rituals	0.57	6. I have compulsive or ritualized hand washing	0.78
7. I check that I did not harm others or that others were not harmed	0.65	**Proportion endorsing at least one symptom**	**0.77**	7. I pick at my skin (obsessions and compulsions)	0.39	7. I have compulsive or ritualized showering, bathing, or toilet routines	0.71
8. I have violent or horrific images in my mind	0.73			**Proportion endorsing at least one symptom**	**0.70**	8. I am overly concerned or disgusted with household items or other inanimate objects	0.83
9. I have obsessions that I might blurt out obscenities or insults	0.73					9. I have compulsions that involve repeated cleaning of household/inanimate objects	0.66
10. I have obsessions that involve doing something else embarrassing	0.72					10. I do other things to prevent or remove contact with contaminants or I avoid doing certain things or going to certain places because of contamination concerns	0.81
11. I obsess about acting on an unwanted impulse	0.82					**Proportion endorsing at least one symptom**	**0.68**
12. I check that nothing terrible will or did happen	0.58						
13. I check or take other measures to prevent or avoid harm coming to myself or others	0.71						
14. I need to repeat routine activities to prevent terrible consequences	0.67						
15. I have mental rituals other than checking	0.77						
**Proportion endorsing at least one symptom**	**0.61**						
**Disease Concerns**		**Sexual Concerns**		**Religious/Moral Concerns**		**Hoarding**	
1. I am obsessed with environmental contaminants (like asbestos, radiation, or toxic waste)	0.79	1. I have forbidden or improper sexual thoughts, images, or impulses	0.92	1. I am obsessed with sacrilege and blasphemy	0.79	1. I have obsessions about needing to save or hoard things for the future	0.95
2. I have mental rituals other than checking related to contamination	0.81	2. I have sexual obsessions that involve children or incest	0.87	2. I am obsessed with what is really right or wrong in a moral sense	0.84	2. I have obsessions about discarding things	0.93
3. I am concerned with illness or disease	0.91	3. I have obsessions about homosexuality	0.72	3. I fear saying certain things	0.88	3. I have obsessions about losing things	0.93
4. I have checking rituals related to obsessions about disease or illness	0.90	4. I have obsessions about violent sexual behavior towards other people	0.93	4. I check to make sure that I have not done anything wrong of a religious nature	0.81	4. I have difficulty deciding whether I should save something or not	0.93
5. I have mental rituals other than checking related to somatic worries	0.86	5. I check to make sure that I have not done anything wrong of a sexual nature	0.76	5. I have compulsions that involve religious duties or objects	0.68	5. I have compulsions to hoard or collect things	0.86
6. I avoid certain actions, people, places or things to prevent obsessions and compulsions about disease from occurring	0.67	6. I avoid certain actions, people, places or things to prevent sexual obsessions and compulsions from occurring	0.86	6. I avoid certain actions, people, places or things to prevent obsessions and compulsions about religion or morality from occurring	0.75	6. I have mental rituals that concern hoarding or saving things	0.85
**Proportion endorsing at least one symptom**	**0.41**	**Proportion endorsing at least one symptom**	**0.24**	7. I need to repeat routine activities to prevent terrible consequences	0.85	7. I avoid certain actions, people, places or things to prevent hoarding compulsions	0.83
				8. I need to tell, ask or confess things	0.81	**Proportion endorsing at least one symptom**	**0.46**
				9. I have mental rituals other than checking related to sexual or religious obsessions	0.85		
				**Proportion endorsing at least one symptom**	**0.46**		
**Body focus**		**Superstition**		**Transformation Concerns**		**Loss/Separation Concerns**	
1. I am excessively concerned with a part of my body or an aspect of my appearance	0.91	1. I have superstitious fears	0.94	1. I am obsessed that I might become a particular person	0.92	1. I obsess about the possibility of being separated from a close family member	0.97
2. I check something related to obsessions about my appearance	1.02	2. I have superstitious behaviors	0.84	2. I have compulsions to rid myself of thinking so much about another person I am obsessed	1.00	2. I have compulsions or rituals that are done in order to prevent the loss of someone (or being separated from someone) very important to me	0.92
3. I have obsessions about food	0.58	3. I have lucky or unlucky numbers	0.88	**Proportion endorsing at least one symptom**	**0.08**	**Proportion endorsing at least one symptom**	**0.39**
4. I have obsessions and/or compulsions about physical exercise	0.69	4. I have obsessions and/or compulsions about colors with special significance	0.75				
**Proportion endorsing at least one symptom**	**0.29**	**Proportion endorsing at least one symptom**	**0.39**				
**Mental/perceptual phenomena**							
1. I avoid certain actions, people, places or things to prevent obsessions and compulsions about symmetry or exactness	0.55						
2. I need to know or remember certain things	0.63						
3. Intrusive nonsense sounds, names, words, or music come into my mind	0.68						
4. Intrusive nonviolent images come into my mind	0.67						
5. I get stuck doing routine behaviors and it slows me down	0.66						
6. I make lists much more than I need to	0.56						
7. I have staring or blinking rituals	0.55						
8. I have the urge to repeat something that I or someone else has said	0.59						
**Proportion endorsing at least one symptom**	**0.64**						

The model is fitted in 60% of the sample.

We next explored ways to fit a more parsimonious model. Decisions to group items under broader factors were based on the degree to which first-order factors were correlated. First, because of a strong correlation between the accuracy, NJR and perceptual/mental factors (*r*s = 0.72, 0.63 and 0.77), we tested a model in which all items included in these factors were grouped under a single incompleteness factor (model 4, Table 1). Second, we grouped all items of the sexual and religious/moral factors (*r* = 0.76) under a single forbidden thoughts factor (model 5, Table 1). Third, all items of the dirt/cleaning and disease factors (*r* = 0.59) were grouped under a contamination factor (model 6, Table 1). The only model that exhibited similar fit as the original model was the model that included a single forbidden thoughts factor. We retained this model because of the principle of parsimony.

**Fig. 1.**
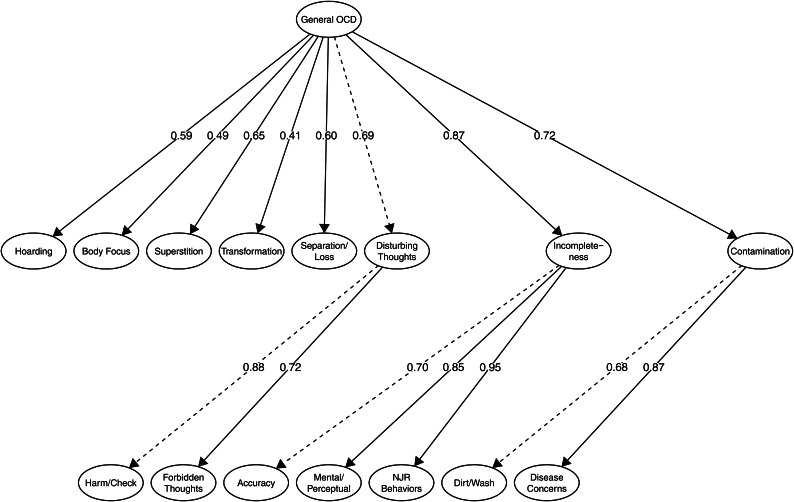
Latent factor model representing empirically derived symptom dimensions of OCD. *Notes.* Item-level data (i.e. indicators) are not shown. Dashed lines indicate which parameter that was fixed in model identification. OCD, obsessive−compulsive disorder; NJR, not just right.

Next, we tested whether we could group substantially correlated first-order factors under higher-order factors and still retain a good model fit. Again, decisions to group factors were based on the degree to which factors were correlated. The forbidden thoughts and harm/check factors (*r* = 0.66) were grouped under an overarching disturbing thoughts factor. Further, the accuracy, perception/mental, and NJR factors (*r*s = 0.72, 0.63 and 0.77) were grouped under an overarching incompleteness factor; and the dirt/wash and disease concerns factors (*r* = 0.59) under an overarching contamination factor. Because of positive correlations among all factors, we also included an overarching, general OCD factor. This parsimonious model exhibited good fit to the data in both 60% of the sample and in the full sample (see models 7 and 8, Table 1). The model is depicted in Fig. 1 and included eight broad symptom dimensions and a general OCD factor.

### Network structure of the symptom dimensions

**Fig. 2.**
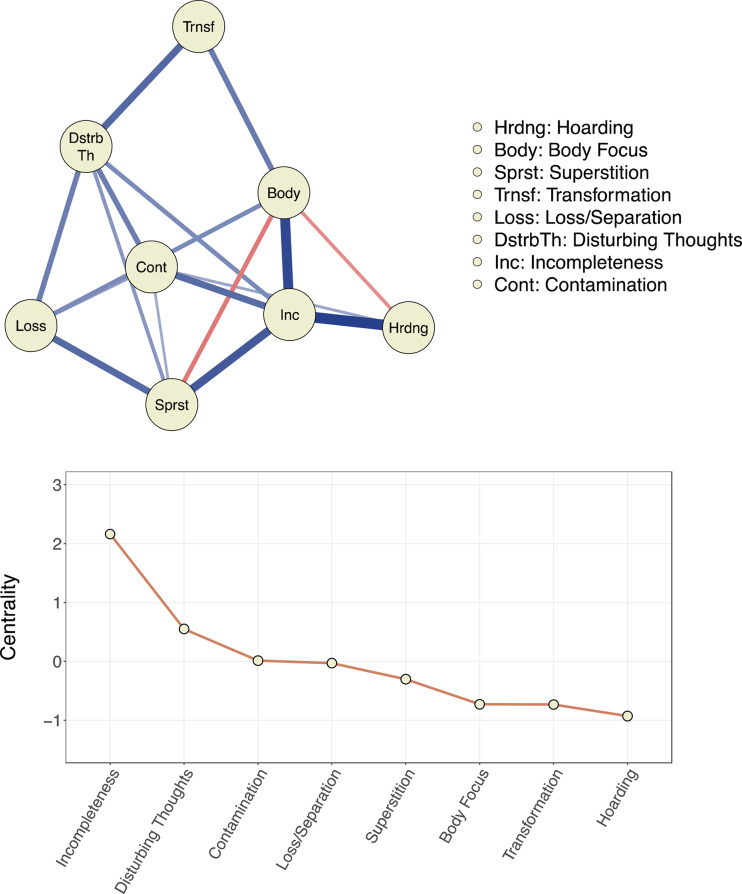
Network model and centrality for empirically derived symptom dimensions of obsessive−compulsive disorder. *Notes.* In the network, symptom dimensions are represented by nodes (circles) and the unique inter-relationship between each symptom dimension pair is depicted as an edge (line). Blue edges indicate positive interconnections. Red edges indicate negative interconnections. For the black and white version of this figure, solid edges indicate positive associations and dashed edges indicate negative associations. Wider and more saturated edges indicate stronger interconnections. Centrality (expected influence) is a numeric estimate for the positive interconnectedness of a specific node; higher values indicate a higher degree of overall interconnectedness. Z-standardized centrality values are presented.

As per our pre-specified analytical plan, the eight broad symptom dimensions outlined above were used as input in the network model. The network structure of the dimensions and expected influence (i.e. centrality) for each dimension are presented in Fig. 2. The incompleteness dimension was statistically significantly more central than all other dimensions. The disturbing thoughts dimension was significantly more central than all other dimensions except the incompleteness dimension. The transformation, hoarding, and body focus dimensions were less central than all other dimensions but not different from each other. Full results for differences in expected influence are in online Supplementary Material Fig. S1.

The results of the separate network analyses for children and adults were broadly similar and presented in online Supplementary Material Fig. S2. The incompleteness dimension was most central in both the child and adult networks and significantly more central than all other dimensions in both networks. In the adult network, the disturbing thoughts dimension was more central than all other dimensions (except the incompleteness dimension). Superstition and disturbing thoughts were highly central in the child network and both dimensions were significantly more central than body focus, hoarding, contamination, and loss/separation.

For the sake of completion, we also present the results of a network analysis based on the less parsimonious 13-factor EFA model (online Supplementary Material Fig. S3). As expected, factors that were grouped in the more parsimonious model (eight dimensions) were closely interconnected in this network.

### Associations with sociodemographic and clinical characteristics

Table 3 shows the results of the SEM regression analyses examining associations between the symptom dimensions and socio-demographic/clinical characteristics. For the *p* factor model, all indicators loaded significantly onto the latent *p* variable.

**Table 3.** Associations (standardized beta coefficients) between socio-demographic/clinical variables and latent symptom dimensions of OCD

	*n*	Symptom dimensions							
		Disturbing thoughts	Incompleteness	Contamination	Hoarding	Body focus	Superstition	Transformation	Loss/Separation
Demographic factors									
Male *v.* female sex	1366	0.18	0.03	−0.19	−0.10	−0.04	0.04	0.03	−0.22*
Current age	1365	0.04	−0.20	0.10	0.29**	0.01	0.17*	−0.26**	−0.10
Child *v.* adult	1365	−0.18	0.10	−0.00	−0.20*	−0.02	−0.40**	0.39**	0.18
OCD factors									
Y-BOCS total	1023	0.11	0.13	0.33**	0.03	0.07	0.04	−0.09	−0.05
Y-BOCS obsessions	1023	0.22*	0.02	0.27**	0.05	0.09	0.06	−0.07	−0.07
Y-BOCS compulsions	1023	0.00	0.22*	0.34**	0.02	0.04	0.02	−0.10	−0.03
Poor insight	967	0.01	−0.00	0.21*	0.06	0.21*	−0.01	−0.20	−0.05
Symptom onset, years	1010	−0.08	−0.25*	0.23*	−0.07	−0.15	−0.09	0.07	−0.04
Progressive course, yes/no	1000	0.26	−0.23	0.20	0.08	0.28**	0.14	−0.25	−0.18
Waxing/waning course, yes/no	1000	−0.22	0.02	−0.00	−0.02	−0.17	−0.01	0.21	0.14
Episodic course, yes/no	1000	0.02	−0.10	−0.04	−0.04	−0.02	−0.01	0.03	−0.10
Plateau course, yes/no	1000	−0.12	0.20	0.10	−0.05	−0.16	−0.17	0.01	0.20
Chronic course, yes/no	1000	−0.08	0.23	−0.10	0.09	−0.13	−0.21	0.07	0.08
Psychiatric comorbidity									
Ongoing Tic disorder	1000	−0.03	0.20	−0.07	0.06	0.06	−0.14	0.18	0.08
Ongoing major depression	1000	0.25*	0.05	−0.02	−0.03	0.11	0.05	−0.10	−0.01
Ongoing social anxiety disorder	1000	0.25*	−0.00	0.14	0.01	0.08	0.05	−0.11	−0.25*
Ongoing generalized anxiety disorder	1000	0.12	−0.02	0.01	−0.02	0.13	0.08	−0.12	0.06
Ongoing panic disorder	1000	0.06	−0.01	0.15	−0.09	−0.25	0.11	0.17	0.09
Past major depression	1000	0.09	−0.12	−0.02	0.11	0.20	0.03	0.08	−0.01
Past anxiety disorder	1000	0.13	0.01	0.03	0.01	0.05	0.01	−0.04	0.07
Lifetime psychosis spectrum	1000	0.18	0.60*	−0.15	−0.06	−0.02	−0.15	0.07	−0.05
Lifetime eating disorder	1000	0.26	0.06	−0.24	−0.03	0.30*	0.12	−0.28	−0.08
Lifetime body dysmorphic disorder	1000	0.01	−0.33	−0.10	0.03	0.98**	0.34**	−0.09	−0.13
Lifetime trichotillomania	1000	−0.23	0.05	0.06	0.07	0.16	−0.18	−0.19	−0.17
Lifetime skin picking disorder	1000	0.01	0.93**	−0.30*	−0.13	−0.14	−0.28*	0.01	0.07
Lifetime illness anxiety disorder	1000	−0.12	−0.52	0.93**	−0.18	0.26	0.09	0.02	−0.01
Self-reported symptoms									
Anxiety symptoms	999	0.24**	0.05	0.12	0.01	0.08	−0.03	0.01	0.11
Depressive symptoms	999	0.26**	0.01	0.01	0.05	0.15	0.05	−0.06	−0.02
Other factors									
Family history OCD	999	0.11	−0.21	0.05	0.22*	0.11	0.12	0.05	−0.19
Family history Tic disorder	927	−0.01	0.34*	−0.13	−0.04	−0.18	−0.01	0.02	0.05
Suicide ideation, lifetime	958	0.32**	0.29*	−0.15	−0.11	0.03	−0.12	0.06	−0.07
Suicide attempt, lifetime	958	0.14	0.49*	−0.22	−0.11	0.02	−0.12	−0.04	−0.00
Sensory phenomena	957	−0.27*	0.89**	0.03	−0.24**	−0.11	−0.21*	−0.01	−0.03
General factor of psychopathology	992	0.11	0.13	−0.10	0.02	0.71**	0.03	−0.04	−0.04

Y-BOCS = Yale−Brown obsessive compulsive scale. OCD = obsessive−compulsive disorder

* indicates *p* < 0.01. ** indicates *p* < 0.001.

Strong positive associations emerged between the incompleteness dimension and lifetime history of SPD, the contamination dimension and lifetime history of IAD, and the body focus dimension and lifetime history of BDD. Because each of the OCD dimensions included items closely related to these comorbid conditions, we reran the analyses and excluded overlapping items. The association between the contamination dimension and IAD was attenuated but significant (*B* = 0.53, *p* < 0.001), as was the association between body focus and BDD (*B* = 0.27, *p* = 0.02). The association between incompleteness and SPD was no longer significant (*B* = 0.03, *p* = 0.82). The body focus dimension was strongly associated with the *p* factor (*B* = 0.71, *p* < 0.001). We reran the *p* factor model but excluded the diagnostic classes related to OCD (i.e. BDD, IAD, SPD) when estimating the *p* factor. The association between the body focus dimension and the *p* factor was attenuated but still significant (*B* = 0.27, *p* < 0.01); no other symptom dimensions were significantly associated with *p* in the model.

## Discussion

We applied state-of-the-art factor and network analytic techniques to shed further light on the most accurate and parsimonious way to conceptualize the heterogeneous symptom nature of OCD using a large multinational sample. Previously well-established symptom dimensions (i.e. forbidden thoughts, symmetry, contamination) replicated but were found to consist of subdimensions of high face validity and theoretical value.

The division of contamination into separate disease concerns and dirt/cleaning dimensions imply that there are multiple mechanisms driving washing and cleaning rituals, such as fear of having an illness and disgust, respectively, which is in line with current evidence (Knowles, Jessup, & Olatunji, 2018). The contamination dimension was associated with a history of IAD, further suggesting that fear of illness may be an important contributor to contamination symptoms among individuals with OCD. Contamination was also associated with more severe OCD and poorer insight which may have clinical relevance as poor insight has been linked to worse long-term outcomes (Catapano et al., 2010). The broad incompleteness dimension was found to consist of three subdimensions: accuracy, perceptual phenomena, and NJR behaviors. These findings highlight the complexity of symmetry-related symptoms in OCD (Jacobsen & Smith, 2017). Furthermore, the incompleteness dimension was strongly associated with a history of suicide attempts, psychosis spectrum disorder and an earlier age at OCD symptom onset, with the latter result replicating previous findings (Katerberg et al., 2010). Prior research had been inconclusive about how to best conceptualize OCD symptoms related to forbidden thoughts (e.g. obsessions about sexual and/or religious themes). We could clarify that such symptoms belong under a separate symptom dimension that is closely related to the dimension that includes thoughts about harm, inflated responsibility, and checking and that these dimensions together form a higher-order disturbing thoughts dimension. As in many previous studies, and the meta-analysis by Bloch et al. (2008), hoarding emerged as a separate dimension.

Findings also outlined four novel or previously underexplored symptom dimensions: body focus, superstition, transformation, and loss/separation. The transformation dimension, endorsed by 8% of participants in our sample, includes what has been described as ‘a fear of turning into someone else or another object or acquiring unwanted characteristics’ (Volz & Heyman, 2007). Others have referred to this symptom class as ‘morphing fears’ which have been theoretically linked to contamination symptoms (Rachman, 2004). Our findings suggest that this dimension is more closely associated with the disturbing thoughts and body focus dimensions than with the contamination dimension, and that transformation symptoms are more common among children/adolescents than among adults (14 and 5%, respectively).

How to best place somatic/body-focused symptoms, which were endorsed by almost a third of all participants in this study (29% of children and 28% of adults), has been a matter of debate in previous studies. Here we show that such symptoms may be best conceptualized as a separate dimension linked to the incompleteness dimension and associated with poor insight, a history of eating disorders and BDD, a late symptom-onset, higher scores on the general *p* factor, and a progressively worsening course of OCD. Given the potential clinical significance of this symptom dimension, more research is needed to fully characterize it and understand its diagnostic boundaries vis-à-vis related disorders, such as BDD and eating disorders.

Superstition has a long tradition in conceptualizations of OCD and other forms of psychopathology (García-Montes, Álvarez, Sass, & Cangas, 2008). In this study, superstitious fears and behaviors, as well as the assignment of special significance to colors and numbers, were endorsed by 39% of participants and emerged as a separate dimension, which was more common in adults than in children (43% *v.* 27%), and associated with a lifetime history of BDD. As the adult version of the Y-BOCS symptom checklist did not include superstitious symptoms, their prevalence may have been previously underestimated in adult populations. Our results show that superstitious symptoms are common, particularly in adults, and should therefore be included in screening tools and be the focus of further study.

Symptoms within the loss/separation dimension were also very common (endorsed by 39% of the participants; 37% of children and 40% adults), and, to the best of our knowledge, has not been previously described as a symptom dimension of OCD. However, a history of separation anxiety disorder is common among individuals with OCD (Mroczkowski et al., 2011). Further, individuals with OCD and comorbid separation anxiety disorder have been shown to have more severe symptoms within the forbidden thoughts dimension (Torres et al., 2016), which is consistent with the unique association between the loss/separation dimension and the forbidden thoughts dimension in this study. It is important to note that this dimension consists of only two items. Future work will be needed to confirm if these items capture a true dimension of OCD or, rather, a co-occurring/comorbid phenomenon.

Of note, previously identified symptom dimensions of OCD have been shown to correspond to compulsive rituals common across normal child development and may reflect processes that have been evolutionarily conserved (Leckman & Bloch, 2008). The four novel symptom dimensions presented here have similar properties and are compatible with an evolutionary and developmental framework of OCD.

Using network analysis, we were able for the first time to quantify the inter-relationships among the symptom dimensions derived from the factor analyses. Incompleteness emerged as the most central dimension (i.e. with the most unique positive associations with other dimensions). In fact, incompleteness had unique positive associations with all other dimensions except loss/separation and transformation and was significantly more central than all other dimensions. This implies that symptoms within this dimension may represent a core phenotype in OCD. The disturbing thoughts dimension was also highly central and uniquely associated with all dimensions except hoarding and body focus. Taken together, these findings are in line with the notion that incompleteness and harm avoidance are the two major emotion-related motivational processes driving compulsive behavior (Summerfeldt, Kloosterman, Antony, & Swinson, 2014). Indeed, the incompleteness dimension was strongly associated with self-reported levels of sensory phenomena which is a construct closely related to the emotion-related process of incompleteness (Prado et al., 2008). Further, the disturbing thoughts dimension was positively associated with self-reported anxiety and a higher likelihood of social anxiety disorder, and many of the OCD symptoms grouped under this dimension have been shown to be motivated by fear and harm avoidance (Cervin, Perrin, Olsson, Claesdotter-Knutsson, & Lindvall, 2020a, 2020b; Ecker & Gonner, 2008). Of note, none of the other dimensions were clearly associated with neither sensory phenomena nor self-reported or diagnosed anxiety, which suggest that other mechanisms may underlie symptom manifestations in these dimensions. Our results regarding the centrality of the incompleteness and disturbing thoughts dimensions imply that these dimensions may represent core broad phenotypes of OCD and more research will be needed to test the hypothesis that targeting these core dimensions in treatment may result in ‘knock-down’ effects on the various sub-dimensions included under each of these core dimensions without necessarily targeting them directly. This hypothesis is empirically testable by analyzing how treatment affects severity across symptom dimensions. If supported, stronger tests could be conducted using randomized-controlled designs.

The hoarding and transformation dimensions were least central in the network. A recent network study of symptom dimensions in pediatric OCD, using another instrument, also found that the hoarding dimension was least central in the symptom dimension network (Cervin et al., 2019a). This is in line with the current classification of hoarding disorder as a mental disorder distinct from OCD (Mataix-Cols & Fernández de la Cruz, 2018), although it is important to remember that difficulties discarding possessions can in some cases be conceptualized as a genuine OCD symptom and treated as such (Pertusa, Frost, & Mataix-Cols, 2010). The transformation dimension was associated with a younger age and, as the proportion of children in the sample was relatively small, this may partly explain the low centrality of this dimension in the full network. Indeed, in the child network, transformation was the fourth most central dimension while it was least central in the adult network.

Prior research based on the Y-BOCS had been inconsistent regarding the structure of OCD symptoms in pediatric and adult samples, potentially due to some important differences in symptom content between the two versions of the scale (e.g. superstitious symptoms are only listed in the child version). In this study, we could overcome such limitations by using the same instrument and found that the symptom structure was largely invariant across the lifespan. Similarly, the network structure of the eight broad dimensions was very similar in the child and adult networks, with the incompleteness dimension being more central than all other dimensions in both networks. Similarities were further emphasized by that the disturbing thoughts dimension was highly central in both networks and that the hoarding dimension was least and second to least central in the child and adult networks, respectively. If our expanded multidimensional model is replicated and supported by future research it can be used to develop more comprehensive screening and assessment tools for OCD that can be employed across the lifespan.

A general OCD factor could be included in the final factor model without a significant decline in model fit according to most fit indices, although a decline in the SRMR index suggested that significant correlations among factors were not represented in this more parsimonious model. A general factor in a structural model will always fit reasonably well when there is substantial observed covariance among included factors, but this does not mean that a single latent mechanism explains all the observed covariance (or even parts of it). Indeed, using network analysis, we could show that there were inter-relationships among the eight broad symptom dimensions that were obscured by attributing all of their overlap to an overarching factor (e.g. the central network position of the incompleteness factor). Taken together, we interpret these findings to be in line with genetically informative studies that show that the etiology of OCD is likely explained by shared genetic and environmental factors that are common to all OCD patients, as well as dimension-specific genetic and environmental risk factors (Iervolino et al., 2011; Taylor, Asmundson, & Jang, 2016). Thus, our expanded multidimensional model could be viewed as a hierarchical model, with different levels of granularity. For some research questions, the optimal level of analysis may be at the general OCD level, whereas more granular levels of analysis may be helpful for other research questions. For example, analyses could focus on the two most central dimensions (incompleteness and disturbing thoughts dimensions) or on the individual symptom dimensions identified here. Researchers can empirically test which of these levels of analysis explains most variance of the desired outcome under study. This line of research could benefit from exploring whether a bifactor model can be fitted to DY-BOCS symptom data as a bifactor model has several statistical advantages over higher-order models.

Some limitations warrant consideration. First, most participants were from Brazil which may affect generalizability as both cross-cultural similarities and differences in OCD symptom expression have been suggested (Nicolini, Salin-Pascual, Cabrera, & Lanzagorta, 2017; Williams, Chapman, Simms, & Tellawi, 2017). Future work should examine whether measurement invariance for the DY-BOCS symptom checklist can be assumed across languages/countries. For such work to be feasible, larger samples are probably needed. Second, although DY-BOCS is the broadest symptom checklist available to date, there may be additional OCD symptoms that are not included. Third, the inclusion of a small set of distinct symptoms (e.g. transformation concerns) may have forced a separate transformation factor to emerge; accordingly, an endless line of factors could potentially emerge given that small sets of distinct and highly correlated symptoms are added. Fourth, many clinical variables were collected retrospectively and as part of routine clinical care, which introduces uncertainties about the validity of these data. Fifth, moderate-to-high correlations among symptom dimensions may have introduced problems with multicollinearity in the SEM regression models causing highly correlated symptom dimensions to exhibit different signs (i.e. negative *v.* positive) in relation to the dependent variable. Finally, the proportion of variance explained by the EFA was modest and somewhat lower than the mean variance explained by EFAs within social science/psychology but this is expected given a large number of binary items (Peterson, 2000).

## Conclusions

There may be a larger number of empirically supported symptom dimensions of OCD than previously thought and some previously established symptom dimensions may consist of subdimensions. The dimensions of incompleteness and disturbing thoughts emerged as most central among these dimensions and may represent core OCD phenotypes. Future research will be needed to establish if this expanded hierarchical and multidimensional model can help improve our understanding of the etiology, neurobiology and treatment of OCD.

## Supplementary material

For supplementary material accompanying this paper visit https://doi.org/10.1017/S0033291720005437. click here to view supplementary material

## References

[ref1] Bloch, M. H., Landeros-Weisenberger, A., Rosario, M. C., Pittenger, C., & Leckman, J. F. (2008). Meta-analysis of the symptom structure of obsessive-compulsive disorder. American Journal of Psychiatry, 165(12), 1532–1542. 18923068 10.1176/appi.ajp.2008.08020320 PMC3972003

[ref2] Cameron, D. H., Streiner, D. L., Summerfeldt, L. J., Rowa, K., McKinnon, M. C., & McCabe, R. E. (2019). A comparison of cluster and factor analytic techniques for identifying symptom-based dimensions of obsessive-compulsive disorder. Psychiatry Research, 278, 86–96. 31163302 10.1016/j.psychres.2019.05.040

[ref3] Catapano, F., Perris, F., Fabrazzo, M., Cioffi, V., Giacco, D., De Santis, V., & Maj, M. (2010). Obsessive–compulsive disorder with poor insight: A three-year prospective study. Progress in Neuro-Psychopharmacology and Biological Psychiatry, 34(2), 323–330. 20015461 10.1016/j.pnpbp.2009.12.007

[ref4] Cervin, M., Perrin, S., Olsson, E., Aspvall, K., Geller, D. A., Wilhelm, S., … Mataix-Cols, D. (2019a). The centrality of doubting and checking in the network structure of obsessive-compulsive symptom dimensions in youth. Journal of the American Academy of Child & Adolescent Psychiatry, 59, 880–889. doi:10.1016/j.jaac.2019.06.018. 31421234 PMC7219532

[ref5] Cervin, M., Perrin, S., Olsson, E., Claesdotter-Knutsson, E., & Lindvall, M. (2019b). Validation of an interview-only version of the Dimensional Yale-Brown Obsessive-Compulsive Scale (DY-BOCS) in treatment-seeking youth with obsessive-compulsive disorder. Psychiatry Research, 271, 171–177. doi:10.1016/j.psychres.2018.11.048. 30481695

[ref6] Cervin, M., Perrin, S., Olsson, E., Claesdotter-Knutsson, E., & Lindvall, M. (2020a). Incompleteness, harm avoidance, and disgust: A comparison of youth with OCD, anxiety disorders, and no psychiatric disorder. Journal of Anxiety Disorders, 69. doi:10.1016/j.janxdis.2019.102175. 31896022

[ref7] Cervin, M., Perrin, S., Olsson, E., Claesdotter-Knutsson, E., & Lindvall, M. (2020b). Involvement of fear, incompleteness, and disgust during symptoms of pediatric obsessive-compulsive disorder. European Child & Adolescent Psychiatry. doi:10.1007/s00787-020-01514-7. PMC7932948 32211970

[ref8] Ecker, W., & Gonner, S. (2008). Incompleteness and harm avoidance in OCD symptom dimensions. Behaviour Research and Therapy, 46(8), 895–904. doi:10.1016/j.brat.2008.04.002. 18514616

[ref9] Epskamp, S., & Fried, E. I. (2018). A tutorial on regularized partial correlation networks. Psychological Methods, 23(4), 617–634. doi:10.1037/met0000167. 29595293

[ref10] Fernandez de la Cruz, L., Micali, N., Roberts, S., Turner, C., Nakatani, E., Heyman, I., & Mataix-Cols, D. (2013). Are the symptoms of obsessive-compulsive disorder temporally stable in children/adolescents? A prospective naturalistic study. Psychiatry Research, 209(2), 196–201. doi:10.1016/j.psychres.2012.11.033. 23261183

[ref11] García-Montes, J. M., Álvarez, M. P., Sass, L. A., & Cangas, A. J. (2008). The role of superstition in psychopathology. Philosophy, Psychiatry, & Psychology, 15(3), 227–237.

[ref12] Goodman, W., Price, L., Rasmussen, S., Mazure, C., Fleischmann, R., Hill, C., … Charney, D. (1989). Yale-Brown obsessive compulsive scale (Y-BOCS). Archives of General Psychiatry, 46, 1006–1011. 2684084 10.1001/archpsyc.1989.01810110048007

[ref13] Iervolino, A. C., Rijsdijk, F. V., Cherkas, L., Fullana, M. A., & Mataix-Cols, D. (2011). A multivariate twin study of obsessive-compulsive symptom dimensions. Archives of General Psychiatry, 68(6), 637–644. 21646580 10.1001/archgenpsychiatry.2011.54

[ref14] Insel, T., Cuthbert, B., Garvey, M., Heinssen, R., Pine, D. S., Quinn, K., … Wang, P. (2010). Research domain criteria (RDoC): Toward a new classification framework for research on mental disorders. American Journal of Psychiatry, 167(7), 748–751. doi:10.1176/appi.ajp.2010.09091379. 20595427

[ref15] Jacobsen, A. M., & Smith, A. J. (2017). Symmetry and ordering in youth with obsessive compulsive disorder. The Wiley Handbook of Obsessive Compulsive Disorders, 1, 405–420.

[ref16] Katerberg, H., Delucchi, K. L., Stewart, S. E., Lochner, C., Denys, D. A., Stack, D. E., … Williams, K. A. (2010). Symptom dimensions in OCD: Item-level factor analysis and heritability estimates. Behavior Genetics, 40(4), 505–517. 20361247 10.1007/s10519-010-9339-z PMC2886912

[ref17] Knowles, K. A., Jessup, S. C., & Olatunji, B. O. (2018). Disgust in anxiety and obsessive-compulsive disorders: Recent findings and future directions. Current Psychiatry Reports, 20(9), 68. 30094516 10.1007/s11920-018-0936-5 PMC6422162

[ref18] Kotov, R., Krueger, R. F., Watson, D., Achenbach, T. M., Althoff, R. R., Bagby, R. M., … Clark, L. A. (2017). The hierarchical taxonomy of psychopathology (HiTOP): A dimensional alternative to traditional nosologies. Journal of Abnormal Psychology, 126(4), 454. 28333488 10.1037/abn0000258

[ref19] Leckman, J. F., & Bloch, M. H. (2008). A developmental and evolutionary perspective on obsessive-compulsive disorder: Whence and whither compulsive hoarding? American Journal of Psychiatry, 165(10), 1229–1233. doi:10.1176/appi.ajp.2008.08060891. 18829875

[ref20] Leckman, J. F., Denys, D., Simpson, H. B., Mataix-Cols, D., Hollander, E., Saxena, S., … Stein, D. J. (2010). Obsessive-compulsive disorder: A review of the diagnostic criteria and possible subtypes and dimensional specifiers for DSM-V. Depression and Anxiety, 27(6), 507–527. doi:10.1002/da.20669. 20217853 PMC3974619

[ref21] Lopez-Sola, C., Fontenelle, L. F., Verhulst, B., Neale, M. C., Menchon, J. M., Alonso, P., & Harrison, B. J. (2016). Distinct etiological influences on obsessive-compulsive symptom dimensions: A multivariate twin study. Depression and Anxiety, 33(3), 179–191. doi:10.1002/da.22455. 26630089 PMC4775288

[ref22] Mataix-Cols, D., & Fernández de la Cruz, L. (2018). Hoarding disorder has finally arrived, but many challenges lie ahead. World Psychiatry, 17(2), 224. 29856565 10.1002/wps.20531 PMC5980544

[ref23] Mataix-Cols, D., Rauch, S. L., Baer, L., Eisen, J. L., Shera, D. M., Goodman, W. K., … Jenike, M. A. (2002). Symptom stability in adult obsessive-compulsive disorder: Data from a naturalistic two-year follow-up study. American Journal of Psychiatry, 159(2), 263–268. 11823269 10.1176/appi.ajp.159.2.263

[ref24] Mataix-Cols, D., Wooderson, S., Lawrence, N., Brammer, M. J., Speckens, A., & Phillips, M. L. (2004). Distinct neural correlates of washing, checking, and hoarding symptom dimensions in obsessive-compulsive disorder. Archives of General Psychiatry, 61(6), 564–576. doi:10.1001/archpsyc.61.6.564. 15184236

[ref25] Miguel, E. C., Ferrão, Y. A., Rosário, M. C. D., Mathis, M. A. D., Torres, A. R., Fontenelle, L. F., … Gonzalez, C. H. (2008). The Brazilian research consortium on obsessive-compulsive spectrum disorders: Recruitment, assessment instruments, methods for the development of multicenter collaborative studies and preliminary results. Brazilian Journal of Psychiatry, 30(3), 185–196. 18833417 10.1590/s1516-44462008000300003

[ref26] Mroczkowski, M. M., Goes, F. S., Riddle, M. A., Grados, M. A., Joseph Bienvenu III, O., Greenberg, B. D., … Murphy, D. L. (2011). Separation anxiety disorder in OCD. Depression and Anxiety, 28(3), 256–262. 21308883 10.1002/da.20773

[ref27] Nicolini, H., Salin-Pascual, R., Cabrera, B., & Lanzagorta, N. (2017). Influence of culture in obsessive-compulsive disorder and its treatment. Current Psychiatry Reviews, 13(4), 285–292. 29657563 10.2174/2211556007666180115105935 PMC5872369

[ref28] Pertusa, A., Fernandez de la Cruz, L., Alonso, P., Menchon, J. M., & Mataix-Cols, D. (2012). Independent validation of the dimensional Yale-Brown obsessive-compulsive scale (DY-BOCS). European Psychiatry, 27(8), 598–604. doi:10.1016/j.eurpsy.2011.02.010. 21570815

[ref29] Pertusa, A., Frost, R. O., & Mataix-Cols, D. (2010). When hoarding is a symptom of OCD: A case series and implications for DSM-V. Behaviour Research and Therapy, 48(10), 1012–1020. 20673573 10.1016/j.brat.2010.07.003

[ref30] Peterson, R. A. (2000). A meta-analysis of variance accounted for and factor loadings in exploratory factor analysis. Marketing Letters, 11(3), 261–275.

[ref31] Prado, H. S., Rosario, M. C., Lee, J., Hounie, A. G., Shavitt, R. G., & Miguel, E. C. (2008). Sensory phenomena in obsessive-compulsive disorder and tic disorders: A review of the literature. CNS Spectrums, 13(5), 425–432. 18496480 10.1017/s1092852900016606

[ref32] Rachman, S. (2004). Fear of contamination. Behaviour Research and Therapy, 42(11), 1227–1255. 15381436 10.1016/j.brat.2003.10.009

[ref33] Robinaugh, D. J., Millner, A. J., & McNally, R. J. (2016). Identifying highly influential nodes in the complicated grief network. Journal of Abnormal Psychology, 125(6), 747. 27505622 10.1037/abn0000181 PMC5060093

[ref34] Rosario-Campos, M. C., Miguel, E. C., Quatrano, S., Chacon, P., Ferrao, Y., Findley, D., … Leckman, J. F. (2006). The dimensional Yale-Brown obsessive-compulsive scale (DY-BOCS): An instrument for assessing obsessive-compulsive symptom dimensions. Molecular Psychiatry, 11(5), 495–504. doi:10.1038/sj.mp.4001798. 16432526

[ref35] Schermelleh-Engel, K., Moosbrugger, H., & Müller, H. (2003). Evaluating the fit of structural equation models: Tests of significance and descriptive goodness-of-fit measures. Methods of Psychological Research Online, 8(2), 23–74.

[ref36] Summerfeldt, L. J., Kloosterman, P. H., Antony, M. M., & Swinson, R. P. (2014). Examining an obsessive-compulsive core dimensions model: Structural validity of harm avoidance and incompleteness. Journal of Obsessive-Compulsive and Related Disorders, 3(2), 83–94. doi:10.1016/j.jocrd.2014.01.003.

[ref37] Taylor, S., Asmundson, G. J., & Jang, K. L. (2016). Etiology of obsessions and compulsions: General and specific genetic and environmental factors. Psychiatry Research, 237, 17–21. doi:10.1016/j.psychres.2016.01.071. 26921046

[ref38] Torres, A. R., Fontenelle, L. F., Shavitt, R. G., Ferrão, Y. A., Do Rosário, M. C., Storch, E. A., & Miguel, E. C. (2016). Comorbidity variation in patients with obsessive–compulsive disorder according to symptom dimensions: Results from a large multicentre clinical sample. Journal of Affective Disorders, 190, 508–516. 26561941 10.1016/j.jad.2015.10.051

[ref39] van den Heuvel, O. A., Remijnse, P. L., Mataix-Cols, D., Vrenken, H., Groenewegen, H. J., Uylings, H. B., … Veltman, D. J. (2009). The major symptom dimensions of obsessive-compulsive disorder are mediated by partially distinct neural systems. Brain, 132(Pt 4), 853–868. doi:10.1093/brain/awn267. 18952675

[ref40] Volz, C., & Heyman, I. (2007). Case series: Transformation obsession in young people with obsessive-compulsive disorder (OCD). Journal of the American Academy of Child & Adolescent Psychiatry, 46(6), 766–772. 17513989 10.1097/chi.0b013e3180465a2e

[ref41] Williams, M. T., Chapman, L., Simms, J., & Tellawi, G. (2017). The Wiley handbook of obsessive compulsive disorders. In J. S. Abramowitz, D. McKay, & E. A. Storch (Eds.), Handbook of obsessive-compulsive disorder across the lifespan. Wiley-Blackwell.

