# Supplementary material for: Towards a definitive symptom structure of obsessive−compulsive disorder: a factor and network analysis of 87 distinct symptoms in 1366 individuals
Source: Psychol Med. 2021 Feb 9;52(14):3267–79. doi: 10.1017/S0033291720005437 (PMC9693708; doi:10.1017/S0033291720005437)
Supplement: Supplementary file 1 [file S0033291720005437sup001.docx]

**Supplemental Data**

**STable 1**. Demographic and clinical characteristics of the different samples.

| Sample | *n* | Sample type | Age in years, *M* (*SD*),  range | Female, *n* (%) | Y-BOCS severity,  *M* (*SD*) | Diagnostic measure |
| --- | --- | --- | --- | --- | --- | --- |
| Brazil 1 | 1001 | Child and adult OCD | 34.85 (12.99), 9-82 years | 569 (56.8%) | 25.51 (7.51) | SCID-CV |
| Brazil 2 | 81 | Child OCD | 11.77 (3.10), 6-17 years | 42 (51.9%) | NA | K-SADS |
| Spain | 95 | Child OCD | 14.87 (2.48), 8-19 years | 46 (48.4%) | NA | K-SADS |
| Turkey | 142 | Child OCD | 12.21 (2.67), 7-18 years | 67 (47.2%) | NA | K-SADS |
| United Kingdom | 47 | Adult OCD | 46.85 (11.38), 25-68 years | 33 (70.2%) | 28.36 (6.62) | MINI |
| Total | 1366 | - | 30.14 (15.01) | 759 (55.5%) | 25.57 (7.50) | - |

*Notes.* Y-BOCS = Yale-Brown Obsessive-Compulsive Scale. OCD = Obsessive-Compulsive Disorder. SCID-CV = Structured Clinical Interview for DSM-IV Axis I Disorders – Clinical Version. K-SADS = Kiddie Schedule for Affective Disorders and Schizophrenia. MINI = Mini International Neuropsychiatric Interview. NA = Not Available.

**STable 2**. Demographic and clinical characteristics of the full sample.

| **Demographic factors** | |  |
| --- | --- | --- |
|  | Female sex, *n* (%) | 759 (55.5%) |
|  | Current age in years, *M* (*SD*) | 30.14 (15.01) |
|  | Child (6-18 years), *n* (%) | 353 (25.9%) |
| **OCD factors** | |  |
|  | Y-BOCS total, *M* (*SD*) | 25.57 (7.50) |
|  | Poor insight, BABS, *M* (*SD*) | 6.85 (5.46) |
|  | Symptom onset, years, *M* (*SD*) | 13.11 (7.80) |
|  | Age at diagnosis, years, *M* (*SD*)  Progressive course, yes, *n* (%)  Waxing/waning course, yes, *n* (%)  Episodic course, yes, *n* (%)  Plateau course, yes, *n* (%)  Chronic course, yes, *n* (%) | 30.96 (12.76)  263 (26.3%)  313 (31.3)  130 (13.0%)  123 (12.3%)  62 (6.2%) |
| **Diagnostic comorbidity, SCID-CV** | |  |
|  | Tic disorder, *n* (%) | 284 (28.4%) |
|  | Major depression, *n* (%) | 332 (33.2%) |
|  | Social anxiety disorder, *n* (%) | 320 (32.0%) |
|  | GAD, *n* (%) | 338 (33.8%) |
|  | Panic disorder, *n* (%) | 113 (11.3%) |
|  | Past Major depression, *n* (%) | 565 (56.4%) |
|  | Past Anxiety disorder, *n* (%) | 657 (65.6%) |
|  | Lifetime Psychosis spectrum, *n* (%) | 29 (2.9%) |
|  | Lifetime Eating disorder, *n* (%) | 114 (11.4%) |
|  | Lifetime Body dysmorphic disorder, *n* (%) | 117 (11.7%) |
|  | Lifetime Trichotillomania, *n* (%) | 60 (6.0%) |
|  | Lifetime Skin picking disorder, *n* (%) | 167 (16.8%) |
|  | Lifetime Illness anxiety disorder, *n* (%) | 34 (3.4%) |
| **Self-reported symptoms** | |  |
|  | Anxiety symptoms, BAI, *M* (*SD*) | 15.94 (11.34) |
|  | Depressive symptoms, BDI, *M* (*SD*) | 16.42 (11.25) |
| **Other factors** | |  |
|  | Family history OCD, yes, *n* (%) | 503 (50.3%) |
|  | Family history Tic disorder, yes, *n* (%) | 181 (19.5%) |
|  | Suicide ideation, lifetime, yes, *n* (%) | 348 (36.3%) |
|  | Suicide attempt, lifetime, yes, *n* (%) | 104 (10.8%) |
|  | Sensory phenomena, USP-SPS, *M* (*SD*) | 4.95 (4.66) |

*Notes.* BABS = Brown Assessment of Beliefs Scale. SCID-I = Structured Clinical Interview for DSM-IV Axis I disorders**.** Y-BOCS = Yale-Brown Obsessive-Compulsive Scale. BAI = Beck Anxiety Inventory. BDI = Beck’s Depression Inventory. USP-SPS = University of São Paulo Sensory Phenomena Scale.

**STable 3.** Factor loadings for all DY-BOCS items in the exploratory factor analysis. Factor loadings > .20 are highlighted in bold. Factor loadings in italics indicate a loading > .20 but that the item is not included in the corresponding factor.

| **Item** | **Harm** | **Dirt/**  **Clean** | **Rel/Moral** | **Hoarding** | **Disease** | **Accuracy** | **NJR Beh** | **Ment/**  **Perc** | **Sexual** | **Loss/**  **Sep** | **Body Focus** | **Superst** | **Transf** | **% Yes in Full Sample** |
| --- | --- | --- | --- | --- | --- | --- | --- | --- | --- | --- | --- | --- | --- | --- |
| Aggressive 1 | **0.59** | -0.09 | 0.00 | -0.04 | 0.09 | 0.11 | 0.16 | ***-0.20*** | -0.01 | -0.01 | -0.02 | 0.00 | 0.18 | 18.1 |
| Aggressive 2 | **0.40** | -0.01 | -0.01 | 0.00 | 0.13 | 0.01 | -0.01 | 0.04 | -0.06 | 0.05 | 0.04 | 0.00 | ***0.24*** | 19.8 |
| Aggressive 3 | **0.42** | 0.08 | 0.11 | -0.04 | 0.05 | 0.00 | 0.01 | -0.05 | -0.09 | -0.10 | 0.00 | -0.04 | ***0.26*** | 7.2 |
| Aggressive 4 | **0.76** | -0.06 | -0.06 | -0.05 | -0.15 | 0.01 | 0.08 | 0.00 | 0.10 | -0.01 | -0.06 | 0.03 | 0.08 | 20.8 |
| Aggressive 5 | **0.69** | -0.07 | -0.04 | 0.07 | 0.00 | 0.05 | -0.11 | -0.02 | 0.04 | -0.07 | -0.03 | 0.00 | 0.02 | 27.2 |
| Aggressive 6 | **0.65** | 0.01 | -0.05 | 0.10 | -0.01 | 0.03 | ***-0.22*** | -0.06 | 0.00 | -0.04 | 0.07 | 0.07 | -0.11 | 26.6 |
| Aggressive 7 | **0.58** | 0.05 | -0.01 | -0.07 | 0.01 | 0.06 | -0.19 | 0.04 | -0.02 | -0.04 | -0.05 | 0.00 | 0.04 | 13.7 |
| Aggressive 8 | **0.51** | 0.02 | -0.12 | 0.08 | -0.04 | -0.16 | -0.02 | 0.17 | 0.14 | **0.21** | -0.05 | 0.05 | -0.07 | 25.2 |
| Aggressive 9 | **0.35** | 0.01 | **0.21** | 0.08 | -0.07 | -0.01 | -0.01 | -0.03 | 0.06 | 0.07 | 0.05 | -0.05 | 0.02 | 13.8 |
| Aggressive 10 | **0.41** | -0.04 | 0.02 | 0.04 | -0.04 | 0.02 | -0.01 | 0.10 | 0.13 | -0.06 | 0.09 | -0.03 | 0.09 | 17.9 |
| Aggressive 11 | **0.63** | 0.02 | -0.16 | 0.04 | -0.08 | -0.03 | 0.07 | -0.01 | ***0.34*** | -0.09 | -0.02 | 0.00 | 0.02 | 16.0 |
| Aggressive 12 | **0.33** | 0.12 | 0.00 | -0.11 | 0.04 | 0.04 | ***-0.20*** | 0.17 | 0.01 | 0.11 | -0.07 | -0.04 | -0.07 | 8.4 |
| Aggressive 13 | **0.61** | 0.09 | 0.02 | 0.00 | 0.00 | 0.01 | -0.06 | -0.07 | -0.09 | -0.02 | -0.03 | -0.06 | 0.01 | 25.1 |
| Aggressive 14 | **0.55** | -0.14 | 0.10 | -0.04 | 0.04 | -0.11 | 0.11 | 0.10 | -0.11 | **0.20** | 0.02 | 0.04 | -0.20 | 26.9 |
| Aggressive 15 | **0.55** | -0.06 | 0.08 | -0.12 | -0.04 | -0.07 | 0.12 | 0.11 | -0.05 | 0.18 | 0.06 | 0.04 | ***-0.23*** | 23.1 |
| Sex/Rel/Moral 1 | 0.01 | 0.05 | 0.12 | 0.05 | 0.09 | -0.05 | 0.08 | -0.03 | **0.73** | -0.01 | 0.02 | -0.03 | 0.00 | 17.3 |
| Sex/Rel/Moral 2 | 0.19 | 0.04 | 0.05 | -0.01 | 0.01 | -0.01 | 0.02 | ***-0.20*** | **0.61** | -0.16 | 0.07 | -0.02 | -0.11 | 6.4 |
| Sex/Rel/Moral 3 | 0.00 | -0.01 | 0.16 | 0.00 | -0.11 | 0.01 | -0.04 | 0.13 | **0.34** | -0.02 | -0.03 | 0.05 | 0.09 | 10.6 |
| Sex/Rel/Moral 4 | 0.10 | -0.04 | -0.15 | 0.00 | 0.12 | -0.07 | ***0.20*** | -0.05 | **0.58** | -0.07 | -0.05 | 0.03 | 0.00 | 5.0 |
| Sex/Rel/Moral 5 | -0.03 | 0.01 | 0.19 | -0.12 | 0.05 | 0.07 | -0.07 | 0.07 | **0.21** | -0.07 | 0.01 | -0.05 | 0.16 | 4.6 |
| Sex/Rel/Moral 6 | -0.11 | 0.09 | ***0.24*** | 0.01 | -0.06 | -0.01 | 0.05 | 0.16 | **0.51** | 0.06 | -0.04 | -0.06 | 0.00 | 9.9 |
| Sex/Rel/Moral 7 | -0.13 | -0.03 | **0.67** | 0.06 | -0.02 | 0.00 | -0.10 | -0.05 | 0.12 | 0.01 | 0.00 | 0.03 | 0.08 | 16.8 |
| Sex/Rel/Moral 8 | 0.10 | 0.01 | **0.60** | 0.04 | 0.09 | 0.09 | -0.19 | 0.05 | -0.01 | 0.14 | -0.01 | 0.05 | -0.08 | 29.5 |
| Sex/Rel/Moral 9 | 0.13 | -0.04 | **0.54** | 0.09 | 0.01 | 0.09 | -0.19 | -0.01 | -0.07 | 0.05 | 0.05 | 0.05 | 0.00 | 22.9 |
| Sex/Rel/Moral 10 | 0.02 | 0.00 | **0.60** | 0.00 | 0.00 | 0.04 | -0.06 | -0.03 | -0.04 | 0.02 | 0.06 | -0.13 | -0.06 | 9.7 |
| Sex/Rel/Moral 11 | 0.03 | -0.05 | **0.55** | -0.01 | -0.04 | 0.00 | 0.18 | -0.18 | 0.00 | 0.05 | -0.08 | 0.12 | -0.05 | 14.2 |
| Sex/Rel/Moral 12 | -0.12 | 0.06 | **0.68** | -0.03 | -0.12 | -0.04 | 0.06 | -0.09 | 0.05 | 0.01 | 0.04 | 0.05 | 0.01 | 14.1 |
| Sex/Rel/Moral 13 | -0.06 | -0.01 | **0.62** | -0.01 | 0.06 | -0.08 | 0.15 | -0.03 | 0.02 | 0.02 | -0.02 | -0.04 | -0.07 | 13.9 |
| Sex/Rel/Moral 14 | 0.04 | -0.02 | **0.59** | -0.05 | -0.04 | -0.07 | -0.09 | 0.02 | 0.01 | 0.07 | 0.04 | 0.02 | 0.06 | 19.9 |
| Sex/Rel/Moral 15 | 0.04 | -0.01 | **0.63** | -0.08 | -0.03 | -0.05 | 0.05 | 0.04 | 0.11 | -0.01 | -0.01 | -0.03 | -0.16 | 19.5 |
| Symmetry 1 | 0.00 | 0.10 | -0.06 | -0.05 | -0.07 | **0.77** | 0.18 | -0.07 | -0.05 | -0.04 | -0.01 | -0.02 | -0.04 | 54.3 |
| Symmetry 2 | -0.02 | -0.01 | 0.01 | -0.05 | -0.03 | **0.75** | ***0.26*** | -0.11 | -0.08 | 0.03 | -0.07 | -0.02 | 0.04 | 40.2 |
| Symmetry 3 | 0.04 | 0.07 | 0.00 | 0.02 | -0.02 | **0.62** | 0.02 | 0.09 | -0.03 | -0.12 | 0.00 | -0.03 | -0.12 | 49.3 |
| Symmetry 4 | 0.04 | 0.09 | 0.03 | 0.06 | 0.03 | **0.48** | -0.06 | 0.08 | -0.04 | -0.16 | 0.05 | 0.06 | -0.17 | 49.3 |
| Symmetry 5 | -0.07 | -0.01 | -0.06 | -0.01 | -0.02 | ***0.26*** | **0.60** | -0.06 | 0.10 | 0.06 | -0.11 | 0.09 | -0.01 | 40.7 |
| Symmetry 6 | 0.05 | -0.02 | -0.09 | 0.02 | 0.03 | ***0.32*** | **0.33** | 0.06 | 0.11 | 0.19 | 0.00 | 0.01 | -0.04 | 25.7 |
| Symmetry 7 | 0.01 | 0.03 | -0.03 | -0.06 | -0.06 | **0.63** | ***0.29*** | -0.02 | -0.07 | 0.02 | -0.03 | -0.02 | -0.07 | 44.6 |
| Symmetry 8 | -0.08 | -0.06 | 0.00 | 0.00 | -0.03 | 0.13 | **0.71** | -0.06 | 0.08 | 0.14 | -0.09 | 0.02 | 0.10 | 24.1 |
| Symmetry 9 | -0.08 | -0.05 | -0.03 | -0.02 | 0.01 | 0.09 | **0.57** | -0.05 | 0.04 | 0.11 | 0.06 | -0.03 | 0.12 | 22.3 |
| Symmetry 10 | 0.10 | -0.01 | 0.10 | 0.09 | 0.03 | **0.30** | 0.02 | 0.01 | 0.14 | -0.06 | 0.11 | 0.07 | -0.08 | 39.0 |
| Symmetry 11 | 0.08 | -0.01 | 0.02 | -0.08 | 0.05 | ***0.20*** | **0.27** | 0.19 | -0.04 | -0.15 | -0.04 | -0.04 | -0.04 | 22.8 |
| Symmetry 12 | -0.02 | 0.04 | 0.08 | 0.00 | -0.04 | 0.14 | 0.15 | **0.31** | -0.06 | -0.11 | 0.01 | 0.06 | -0.10 | 24.7 |
| Contamination 1 | 0.00 | **0.81** | -0.06 | 0.02 | -0.02 | 0.08 | -0.10 | -0.09 | 0.07 | -0.05 | 0.06 | 0.00 | -0.07 | 40.8 |
| Contamination 2 | -0.02 | **0.83** | 0.04 | 0.02 | -0.17 | -0.02 | -0.10 | 0.08 | 0.06 | 0.10 | -0.02 | 0.00 | -0.04 | 33.9 |
| Contamination 3 | 0.05 | ***0.25*** | 0.01 | 0.03 | **0.27** | -0.01 | 0.03 | -0.06 | -0.12 | -0.11 | -0.02 | 0.05 | ***0.26*** | 11.9 |
| Contamination 4 | 0.00 | **0.48** | 0.03 | 0.02 | 0.17 | 0.03 | 0.01 | -0.11 | -0.01 | 0.01 | 0.06 | 0.02 | 0.11 | 25.2 |
| Contamination 5 | -0.06 | **0.38** | 0.00 | 0.00 | 0.08 | 0.07 | 0.05 | 0.08 | -0.01 | 0.12 | 0.00 | -0.02 | 0.06 | 13.7 |
| Contamination 6 | -0.01 | **0.44** | 0.03 | 0.04 | ***0.39*** | -0.02 | -0.08 | -0.12 | 0.06 | 0.12 | -0.09 | 0.06 | 0.00 | 31.3 |
| Contamination 7 | -0.02 | **0.74** | -0.03 | -0.01 | -0.10 | 0.05 | 0.07 | -0.10 | 0.08 | -0.18 | -0.03 | 0.04 | -0.01 | 43.6 |
| Contamination 8 | 0.03 | **0.63** | 0.00 | -0.02 | -0.07 | 0.04 | 0.04 | -0.05 | -0.05 | -0.06 | 0.05 | 0.03 | -0.14 | 37.8 |
| Contamination 9 | -0.04 | **0.78** | -0.01 | -0.01 | -0.01 | -0.04 | 0.04 | 0.10 | 0.06 | 0.12 | -0.09 | -0.02 | 0.07 | 33.3 |
| Contamination 10 | -0.02 | **0.68** | -0.02 | 0.02 | -0.15 | 0.17 | -0.06 | 0.05 | -0.08 | 0.08 | -0.02 | 0.06 | -0.05 | 32.1 |
| Contamination 11 | 0.08 | ***0.29*** | 0.03 | -0.06 | **0.33** | -0.13 | 0.15 | -0.02 | 0.08 | -0.10 | 0.01 | -0.11 | 0.05 | 14.3 |
| Contamination 12 | 0.06 | **0.69** | -0.04 | 0.01 | 0.00 | -0.09 | -0.14 | 0.17 | -0.02 | -0.04 | -0.04 | 0.03 | -0.10 | 35.5 |
| Hoarding 1 | -0.01 | 0.07 | -0.01 | **0.93** | -0.01 | -0.03 | -0.10 | -0.04 | 0.05 | 0.01 | -0.01 | 0.07 | -0.11 | 35.9 |
| Hoarding 2 | 0.00 | 0.05 | -0.02 | **0.86** | 0.02 | -0.03 | 0.02 | -0.06 | 0.00 | 0.10 | -0.05 | 0.08 | -0.05 | 33.5 |
| Hoarding 3 | -0.03 | 0.05 | -0.10 | **0.68** | 0.04 | 0.01 | 0.05 | 0.06 | 0.04 | 0.03 | 0.00 | -0.10 | -0.07 | 22.8 |
| Hoarding 4 | 0.00 | -0.06 | 0.02 | **0.77** | 0.04 | 0.02 | -0.08 | 0.02 | 0.00 | -0.02 | 0.06 | 0.04 | -0.05 | 33.6 |
| Hoarding 5 | 0.04 | -0.01 | -0.03 | **0.82** | -0.07 | 0.02 | 0.01 | -0.09 | -0.04 | -0.02 | -0.09 | 0.06 | 0.00 | 28.9 |
| Hoarding 6 | -0.01 | -0.02 | 0.07 | **0.31** | 0.03 | -0.04 | ***0.24*** | 0.14 | 0.06 | -0.05 | -0.12 | -0.17 | 0.15 | 7.3 |
| Hoarding 7 | 0.00 | -0.01 | 0.17 | **0.33** | -0.05 | -0.11 | 0.08 | 0.13 | -0.13 | -0.15 | -0.05 | 0.00 | 0.12 | 7.6 |
| Miscellaneous 1 | -0.06 | -0.04 | -0.05 | 0.04 | **0.90** | 0.01 | -0.09 | -0.09 | 0.09 | ***0.24*** | 0.05 | 0.08 | -0.05 | 26.6 |
| Miscellaneous 2 | -0.02 | -0.06 | -0.03 | -0.02 | **0.80** | 0.06 | -0.05 | -0.14 | -0.02 | 0.14 | 0.03 | 0.04 | 0.07 | 16.9 |
| Miscellaneous 3 | -0.04 | -0.04 | -0.02 | 0.03 | **0.78** | -0.10 | 0.02 | 0.10 | 0.05 | **0.20** | -0.04 | -0.08 | -0.06 | 11.2 |
| Miscellaneous 4 | -0.01 | -0.01 | -0.09 | -0.04 | **0.57** | -0.07 | 0.00 | 0.03 | 0.01 | 0.12 | 0.11 | 0.13 | 0.00 | 11.0 |
| Miscellaneous 5 | 0.11 | -0.03 | -0.07 | 0.08 | 0.00 | 0.06 | -0.06 | **0.55** | -0.05 | 0.02 | 0.00 | -0.05 | 0.11 | 16.8 |
| Miscellaneous 6 | -0.02 | 0.02 | 0.00 | 0.09 | 0.17 | -0.01 | -0.02 | 0.10 | -0.01 | 0.00 | 0.04 | **0.74** | 0.10 | 25.9 |
| Miscellaneous 7 | 0.02 | 0.11 | -0.04 | 0.01 | 0.03 | 0.00 | -0.05 | 0.10 | -0.05 | -0.09 | 0.03 | **0.76** | 0.08 | 19.3 |
| Miscellaneous 8 | -0.03 | -0.02 | 0.10 | -0.01 | 0.07 | -0.03 | ***0.31*** | 0.02 | 0.13 | -0.08 | 0.03 | **0.46** | 0.00 | 22.9 |
| Miscellaneous 9 | 0.07 | 0.02 | **0.20** | -0.01 | -0.03 | 0.00 | 0.17 | 0.14 | -0.07 | 0.00 | -0.15 | **0.39** | 0.14 | 14.1 |
| Miscellaneous 10 | 0.00 | -0.02 | -0.05 | -0.03 | 0.01 | 0.04 | 0.05 | **0.56** | 0.11 | 0.19 | -0.02 | 0.02 | 0.17 | 23.4 |
| Miscellaneous 11 | 0.03 | 0.02 | -0.07 | -0.05 | -0.13 | -0.07 | ***-0.21*** | **0.83** | -0.03 | 0.03 | -0.04 | 0.13 | ***0.20*** | 14.0 |
| Miscellaneous 13 | -0.10 | 0.13 | 0.03 | 0.02 | 0.10 | -0.02 | 0.19 | **0.30** | 0.03 | -0.04 | 0.06 | -0.06 | 0.01 | 29.6 |
| Miscellaneous 14 | -0.03 | -0.09 | -0.05 | 0.15 | 0.07 | ***0.21*** | -0.07 | **0.23** | 0.03 | 0.02 | 0.06 | -0.04 | 0.00 | 15.7 |
| Miscellaneous 15 | 0.05 | 0.02 | 0.10 | 0.01 | ***0.29*** | -0.04 | ***0.20*** | 0.15 | -0.09 | **0.81** | -0.05 | -0.04 | ***-0.26*** | 35.5 |
| Miscellaneous 16 | 0.06 | 0.01 | 0.12 | 0.00 | ***0.31*** | -0.07 | ***0.30*** | 0.02 | -0.11 | **0.77** | -0.10 | -0.05 | ***-0.28*** | 29.1 |
| Miscellaneous 17 | 0.05 | -0.01 | -0.07 | 0.00 | -0.01 | -0.05 | 0.09 | 0.15 | 0.02 | ***-0.22*** | 0.04 | 0.06 | **0.55** | 4.2 |
| Miscellaneous 18 | 0.00 | -0.06 | 0.00 | -0.08 | -0.02 | -0.12 | 0.19 | ***0.29*** | -0.01 | -0.11 | -0.07 | 0.04 | **0.62** | 6.7 |
| Miscellaneous 19 | 0.11 | -0.07 | 0.00 | -0.01 | 0.00 | 0.09 | 0.18 | **0.32** | -0.13 | 0.02 | 0.02 | -0.01 | 0.15 | 14.9 |
| Miscellaneous 20 | -0.09 | 0.05 | -0.05 | -0.01 | 0.01 | -0.05 | 0.08 | **0.36** | 0.08 | 0.03 | 0.17 | 0.04 | 0.05 | 15.1 |
| Miscellaneous 21 | -0.03 | 0.04 | 0.02 | -0.03 | -0.07 | -0.04 | -0.07 | ***0.26*** | -0.06 | 0.01 | **0.70** | -0.02 | 0.06 | 21.4 |
| Miscellaneous 22 | -0.05 | -0.01 | 0.09 | -0.10 | 0.06 | 0.01 | -0.08 | 0.09 | -0.05 | -0.02 | **0.85** | -0.08 | 0.07 | 19.3 |
| Miscellaneous 23 | -0.01 | -0.04 | -0.01 | 0.03 | 0.08 | 0.02 | -0.04 | -0.04 | -0.03 | -0.06 | **0.54** | 0.02 | -0.04 | 9.4 |
| Miscellaneous 24 | -0.01 | -0.05 | 0.01 | -0.04 | 0.06 | -0.03 | -0.01 | -0.06 | 0.07 | -0.10 | **0.48** | 0.05 | -0.05 | 4.5 |
| Miscellaneous 25 | -0.07 | 0.02 | 0.03 | -0.01 | -0.01 | 0.04 | **0.26** | ***0.20*** | -0.09 | 0.10 | 0.16 | 0.05 | 0.06 | 13.2 |
| Miscellaneous 26 | 0.02 | 0.08 | -0.04 | 0.01 | -0.13 | -0.05 | 0.16 | -0.02 | -0.02 | 0.05 | 0.09 | -0.01 | 0.03 | 5.8 |
| Miscellaneous 27 | 0.13 | 0.05 | 0.07 | 0.08 | -0.04 | 0.03 | **0.20** | -0.15 | -0.01 | 0.13 | 0.15 | -0.04 | 0.07 | 19.5 |

*Notes.* DY-BOCS = Dimensional Yale-Brown Obsessive Compulsive Scale.

**STable 4.** Fit indices for the 13-factor model in subsamples.

|  | *n* | *χ2* | | *df* | | *p* | | CFI | | TLI | | RMSEA | | SRMR | |  |
| --- | --- | --- | --- | --- | --- | --- | --- | --- | --- | --- | --- | --- | --- | --- | --- | --- |
| Children/adolescents | 355 | | 3887.70 | | 3491 | | < .001 | | .937 | | .934 | | .018 | | .120 | |
| Adults | 1012 | | 5772.91 | | 3491 | | < .001 | | .939 | | .937 | | .025 | | .082 | |
| Men | 609 | | 4328.73 | | 3407 | | < .001 | | .948 | | .946 | | .021 | | .089 | |
| Women | 759 | | 4995.48 | | 3491 | | < .001 | | .936 | | .933 | | .024 | | .091 | |
| Brazil | 1082 | | 5790.74 | | 3491 | | < .001 | | .941 | | .938 | | .025 | | .080 | |
| Europe | 286 | | 3870.01 | | 3491 | | < .001 | | .929 | | .925 | | .020 | | .135 | |

*Notes.* DY-BOCS = Dimensional Yale-Brown Obsessive Compulsive Scale. *χ2* = Chi-Squared*. df* = Degrees of Freedom. CFI = Comparative Fit Index. TLI = Tucker-Lewis Index. RMSEA = Root Mean Square Error of Approximation. SRMR = Standardized Root Mean Square Residual. Best fitting model, because of the principle of parsimony, highlighted in bold. One hoarding item was omitted when fitting the model to the data for men because of a very high correlation between this item and another hoarding item.

**SFigure 1.** Statistically significant differences in Expected Influence for the different symptom dimensions in the network structure of these dimensions.

*Notes.* Black boxes indicate a significant difference. Point estimates of Expected Influence for each node are in the diagonal. Nodes are sorted according to their Expected Influence estimate with the node with the highest estimate at the top of the y-axis. Inc = Incompleteness. DstrbTh = Disturbing Thoughts. Cont = Contamination. Loss = Separation/Loss. Sprst = Superstition. Trnsf = Transformation. Hrdng = Hoarding.

**SFigure 2.** Network structures for children and adolescents and Expected Influence for the dimensions in each network.

*Notes.* Black boxes indicate a significant difference. Point estimates of Expected Influence for each node are in the diagonal. Nodes are sorted according to their Expected Influence estimate with the node with the highest estimate at the top of the y-axis. Inc = Incompleteness. DstrbTh = Disturbing Thoughts. Cont = Contamination. Loss = Separation/Loss. Sprst = Superstition. Trnsf = Transformation. Hrdng = Hoarding. Body = Body Focus.

**SFigure3.** Network structure of the 13 factors derived from exploratory factor analysis fitted in the full sample.

*Notes.* NJR = Not Just Right. Accuracy = Accuracy. Mental = Mental/Sensory. Dirt = Dirt/Cleaning. Dseas = Disease Concerns. Sex = Sexual Concerns. Rel = Religious/Moral Concerns. Loss = Separation/Loss. Sprst = Superstition. Trnsf = Transformation. Hrdng = Hoarding. Body = Body Focus.
